# Granular Cell Tumor of the Pituitary Stalk: A Rare and Benign Entity

**DOI:** 10.5334/jbr-btr.841

**Published:** 2015-09-15

**Authors:** A. Gregoire, P. Bosschaert, C. Godfraind

**Affiliations:** 1 Department of Radiology, Clinique St-Pierre, Ottignies, Belgium; 2 Department of Pathology, Cliniques Universitaires St-Luc, Bruxelles, Belgium

**Keywords:** Pituitary, neoplasms

## Abstract

Granular cell tumor of the pituitary stalk is an uncommon benign lesion that usually appears like a suprasellar mass with visual disturbances. This diagnosis should be considered with the discovery of neurohypophysis tumors. Histological confirmation remains essential. We describe one case of this rare entity and review the literature.

## Case report

**Figure 1:**
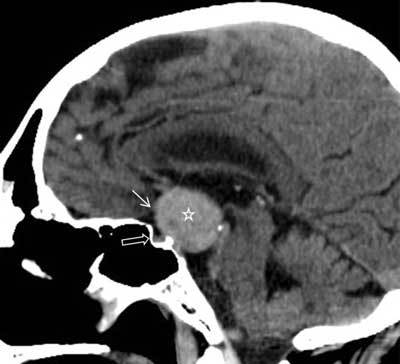
Sagittal non-enhanced CT image showing a spontaneously well circumscribed hyperdense suprasellar mass (asterisk) near the optic chiasm (arrow). The pituitary gland is not involved (open arrow).

**Figure 2:**
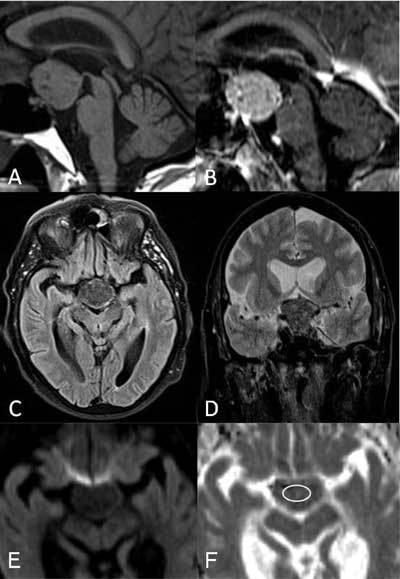
Sagittal T1-weighted MR images before (A) and after (B) gadolinium administration demonstrating a somewhat inhomogeneous aspect with moderate to intense enhancement of the tumor. Axial flair (C) and coronal T2-weighted (D) images showing the occupation of the opto-chiasmatic cistern and the low signal intensity to gray matter of the lesion. Diffusion-weighted image (E) demonstrating a slight hypointensity with ADC value estimated at 0.70 × 10^−3^ mm^2^/s (F)(circle).

A 68-year-old man was examined for bitemporal hemianopsia and falling episodes. Computed tomography (CT) revealed a 32 mm spontaneously hyperdense suprasellar mass (Fig. 1). No calcification was noted. Complementary preoperative magnetic resonance imaging (MRI) showed a well delineated lesion with a nearly isointense signal on the T1-weighted images and a low signal intensity on the T2-weighted images. T1-weighted images following gadolinium administration demonstrated an inhomogeneous moderate to intense enhancement. Diffusion-weighted images showed a slight hypointensity with apparent diffusion coefficient (ADC) measured at 0.70 × 10^−3^ mm^2^/s (Fig. 2). The adjacent pituitary gland was estimated as normal. Meningioma was the first diagnostic hypothesis.

A surgical intervention was planned but only subtotal resection was possible because of the lesion attachment to the chiasm and optic nerves. Histological examination provided the diagnosis of granular cell tumor (GCT). The postoperative course was uneventful and the patient’s progress was satisfactory. No evidence of recurrence has been identified after two years of observation.

## Discussion

GCT of the neurohypophysis was first described by Boyce and Beadles in 1893 [1]. A morphological study published in 1955 by Luse and Kernohan [2] revealed that minute granular cell nests or pinhead-sized granular cell tumorettes were observed in 6.4% of autopsies realized in asymptomatic patients, most commonly in older patients, with a same frequency in the pituitary stalk and the post-hypophysis. We note that most of the reported cases with available imaging show suprasellar or intra- and suprasellar locations but almost never purely intrasellar.

Generally, this is a benign, slow growing and non-secreting tumor that does not show any space-occupying effect when it is small. For the largest lesions, symptoms of compression can appear as visual disturbances (in 70% of cases) or headaches [3]. Surprisingly, hypothalamo-hypophysar dysfunction was observed in only a few patients [4]. This kind of lesion occurs with a peak incidence in the fifth decade of life and affects women with more frequency (2:1), black women in particular. GCTs represent less than 0.1% of all primary brain tumors. To date, forty-six symptomatic cases are reported [5].

Apart from the particular location, this lesion does not show specific characteristics. It is a well circumscribed, rounded retrochiasmatic mass, hyperdense on non-enhanced CT. MRI findings show a usual isointense signal on T1-weighted images and an iso- or hypointense signal on T2-weighted images. Heterogeneous enhancement is found in twelve of the twenty-three described cases with contrast agent use. Others enhance homogeneously [5, 6]. Regarding the largest tumors, no enlargement or either destruction of the sella has been described [7]. Presence of calcification might depend on lesion size [3, 7, 8].

Depending on the lesion size, trans-sphenoidal or temporal approach is selected [7]. The aim of the surgery is two-fold: first, decompress the optic pathways, and second, obtain a histologic diagnosis. Surgical removal is quite difficult and only subtotal removal is often possible in most of cases because the high vascularization and the close connection to the optic chiasm. Maximal resection involves the removal of the stalk leading to a possible irreversible hypopituitarism.

Regrowth after resection have been reported, therefore some authors advised postoperative radiotherapy after subtotal removal, but it is still polemic. Malignant GCTs are extremely rare and none have been reported arising from the neurohypophysis [3].

**Figure 3:**
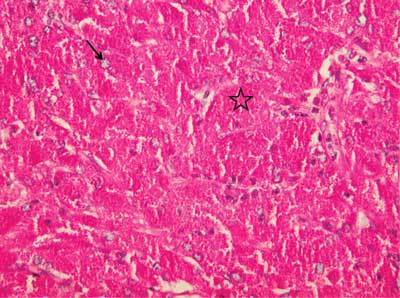
Tumor constituted of polygonal cells presenting a granular cytoplasm and bland ovoid nuclei (arrow). PAS (diastase digestion) reveals the positivity of the cytoplasmic granules (asterisk).

The histological examination revealed tumors composed of large and irregularly polyhedral cells, with a round nucleus, delicate chromatin, and uniform nucleoli. The most typical feature is an abundance of periodic acid-Schiff (PAS) positive granules in the ample cytoplasm. Immuno-histochemical findings vary. Some studies report a positive nuclear reaction for S-100 protein and glial fibrillary acidic protein (GFAP) which is normally expressed in the pituityctes. Both are positive in our case. Mitotic activity is here minimal, and necrosis is not seen [9, 10] (Fig. 3).

The cellular origin of the GCTs remains uncertain. Many possible origins have been described [3]. Some authors consider that these tumors arise from modified glial cells originating from ependyma called pituicytes. It would therefore explain the specific localization on the pituitary stalk. 5 types of pituicytes have been identified and classified as a progenitor of a separate type of neurological tumor, such as granular cells, major cells, dark cells, oncocytic cells, and ependymal cells. Most authors agree that the granular cell tumors arise from granular cell pituicytes [9, 10] and are different from their extracranial counterparts [3, 4, 11].

The differential diagnosis of primary nonadenomatous pituitary gland tumors includes 2 other entities: pituicytoma and spindle cell oncocytoma. Pituicytoma is a purely intrasellar lesion, iso-intense to gray matter on T1-weighted images and hyperintense on T2-weighted images, and enhances homogeneously after gadolinium administration. Spindle cell oncocytoma is a mixed supra and intrasellar tumor that cannot be separated from the pituitary gland, and also presents a homogeneous enhancement after gadolinium administration. A panhypopituitarism is more common [5]. More broadly, we must also mention other local conditions like pituitary gland adenoma, the most common pituitary mass, sometimes with suprasellar component, pituitary stalk meningioma, metastase from extracranial primary source, Langerhans cell histiocytosis, germinoma, lymphoma, glioma and craniopharyngioma [3]. Finally, aneurysm, teratoma and chordoma should also be considered [7].

In conclusion, the lesion we described here is rare but nevertheless should be included in the differential diagnosis of a suprasellar mass, especially if well circumscribed, and in conjunction with visual disturbances.

## Competing Interests

The authors declare that they have no competing interests.

## References

[1] Boyce R, Beadles CF (1993). A further contribution to the study of the pathology of the hypophysis cerebri. J Pathol Bacteriol.

[2] Luse S, Kernohan J (1955). Granular cell tumors of the stalk and posterior lobe of the pituitary gland. Cancer.

[3] Saiegh L, Odeh M, Sheikh-Ahmad M (2013). Granular cell tumor of the neurohypophyse: case report and review of the literature. Neuroendocrinol Lett.

[4] Menon G, Easwer H, Radhakrishnan V (2008). Symptomatic granular cell tumour of the pituitary. Br J Neurosurg.

[5] Covington M, Chin S, Osborn A (2011). Pituicytoma, spindle cell oncocytoma and granular cell tumor: clarification and mete-analysis of the world literature since 1893. Am J Neuroradiol.

[6] Iglesias A, Arias M, Brasa J (2000). MR imaging findings in granular cell tumor of the neurohypophysis: a difficult preoperative diagnosis. Eur Radiol.

[7] Buhl R, Hugo H, Hempelmann R (2001). Granular-cell tumour: a rare suprasellar mass. Neuroradiol.

[8] Schaller B, Kirsch E, Tolnay M (1998). Symptomatic granular cell tumor of the pituitary gland: case report and review of the literature. Neurosurg.

[9] Vaquero J, Leunda G, Cabezudo J (1981). Granular pituicytomas of the pituitary stalk. Acta Neurochir.

[10] Nishio S, Takeshita I, Yoshimoto K (1998). Granular cell tumor of the pituitary stalk. Clin Neurol Neurosurg.

[11] Ji C, Teng M, Chang T (1995). Granular cell tumour of the neurohypophysis. Neuroradiol.

